# Aquaporin and Blood Brain Barrier

**DOI:** 10.2174/157015910791233132

**Published:** 2010-06

**Authors:** Bonomini Francesca, Rita Rezzani

**Affiliations:** Division of Human Anatomy, Department of Biomedical Sciences and Biotechnologies, University of Brescia, V.le Europa 11, 25123 Brescia, Italy

**Keywords:** Central nervous system, blood brain barrier, aquaporin.

## Abstract

Large water fluxes continuously take place between the different compartments of the brain as well as between the brain parenchyma and the blood or cerebrospinal fluid.

Disturbances in this well-regulated water homeostasis may have deleterious effects on brain function and may be fatal in cases where water accumulates in the brain following pathologies such as ischemia, haemorrhage, or brain trauma.

The molecular pathways by which water molecules cross the blood brain barrier are not well-understood, although the discovery of Aquaporin 4 (AQP4) in the brain improved the understanding of some of these transport processes, particularly under pathological conditions.

## 1. AQUAPORINS AND BLOOD BRAIN BARRIER

AQP1 and AQP4 are known to be expressed in the central nervous system (CNS), and it is possible that these proteins contribute to water transport across the blood brain barrier (BBB).

### 1.1. Aquaporin 1

In the CNS, AQP1 was demonstrated only in the epithelium (but not in the fenestrated capillaries) of the choroid plexus and in the ependyma and pia [29]. Nevertheless, expression of AQP1 was not detected in freshly isolated rat cerebral microvessels, as determined by reverse-transcriptase–polymerase chain reaction (RT–PCR) and western blotting [18]. The brain endothelial phenotype differs from that of many other tissues; *in vivo* it is maintained by the influence of the apposing astrocytic end-feet, but some features can be lost *in vitro* [15, 25, 26]. However, aspects of the phenotype may be up-regulated when the cells are co-cultured with astrocytes [1]. The apparent lack of AQP1 expression in brain endothelial cells may represent an important phenotypic difference among these and the endothelia of many other tissues which support expression of AQP1, as determined by immunocytochemistry [29].

Recently, it has been demonstrated that primary cultured rat brain microvessel endothelial cells express only very low levels of AQP1, in agreement with the findings that AQP1 is not detectable in cerebral microvessels in situ or when freshly isolated [18, 29]. However, AQP1 expression has been showed to be greatly up-regulated in passaged cultured rat brain microvessel endothelial cells, conditions under which de-differentiation is known to occur. In addition, the Authors demonstrated that AQP1 mRNA levels can be reduced by co-culture with astrocytes, implicating astrocytic factors in the control of AQP1 expression [16].

*In vivo* the brain endothelial phenotype is induced and maintained by the influence of astrocytes; some features, such as expression of γ-glutamyl transpeptidase, can be lost in passaged cells but are retained or up-regulated in co-cultures with astroglial cells [1]. The reverse occurs with AQP1: AQP1 mRNA is up-regulated with repeated passaging and decreased by co-culture with astrocytes. This supports the hypothesis that AQP1 expression is suppressed by astrocytic factors; on the contrary, when this astrocytic influence is absent, AQP1 expression increases.

In a study of AQP1 expression in human brain, a small number of microvessels were positively stained, but there was marked up-regulation in endothelium in astrocytomas and metastatic carcinomas; BBB function is known to be impaired in such brain tumours, leading to formation of edema [35]. Down-regulation of the tight-junction proteins claudin and occludin has also been demonstrated in microvessels in glioblastoma multiforme [19]. Thus, loss of barrier function and the expression of AQP1 may both be regarded as down-regulation of BBB phenotype. It is not possible to say whether this is caused by loss of normal astrocyte function; in fact, an increase in microvessel permeability could arise from overexpression of vascular endothelial growth factor (VEGF) in such tumours [34]. However, although it is well established that astrocytic factors are important in maintaining the tightness of BBB, Dolman and co-workers [16] showed that they play a role in reduction of endothelial AQP1 expression [1]. This may provide an explanation for up-regulation of endothelial AQP1 in tumours involving astroglial cells.

### 1.2. Aquaporin 4

Given its polarized expression bordering the BBB and the brain–cerebrospinal fluid interface, AQP4 has been presumed to play an important functional role in the transport of water in and out of the brain parenchyma by enhancing transmembrane water flux in astrocytes [38] Fig. (**1**). Solenov and colleagues [39] demonstrated that astrocytes lacking AQP4 had sevenfold reduced water permeability, as compared with equivalent cells with normal AQP4 expression. The functional importance of AQP4-facilitated water transport in diseases of cerebral water imbalance has been primarily tested *in vivo* using mice with altered or absent AQP4 expression [12].

The lack of an apparent phenotype of the AQP4 knockout mice also suggested that the role of AQP4 under normo-physiological conditions might be limited [20]. However, during pathological conditions leading to the formation of brain edema, AQP4 is an important player. Knock-out of AQP4 or disruption of its polarized expression pattern mitigated the brain water accumulation associated with brain ischemia, water intoxication, and hyponatremia [3, 4, 6, 22, 47], suggesting that during pathophysiological conditions, AQP4 is a main entrance route for water from the plasma and into the brain.

The role of AQP4 in CNS water balance is now well characterized. AQP4 deficiency reduced cytotoxic edema produced by water intoxication, cerebral hischemia and acute bacterial meningitis [22, 32], but increases vasogenic edema caused by brain tumour, cortical freeze injury, brain abscess and kaolin-induced obstructive hydrocephalus [11, 13, 31].

## 2. AQUAPORINS AND BRAIN EDEMA

Cerebral edema is due to abnormally increased water content and consequent brain swelling in different pathophysiological pathway of CNS vascular diseases.

The recent identification of AQPs has consented to understand the basis of the water transport in many tissues, including CNS. Since brain edema continues to be the main cause of death from several CNS diseases, including brain infarct, haemorrhage, tumour, trauma and infection, the interest in AQPs and their functional contribution to the water balance is due to their possible therapeutic role in managing brain edema [2, 5, 10, 22]. Data on AQP expression in cerebral edema, particularly AQP1 and AQP4, which are expressed in the CNS and participate in water transport across the BBB, come from some recent studies of large ischemic strokes [9, 16, 43].

The presence of AQP4 at the BBB suggests that it is important for the brain water balance and may play a critical role in brain edema [28]. AQP4 over-expression in human astrocytomas correlates with detection of brain edema on magnetic resonance imaging [35, 45]. However, under-expression of AQP4 protein is associated with early stage edema in rodents subjected to permanent focal brain ischemia and hypoxia-ischemia [14, 24]. In traumatic brain injury AQP4 mRNA is decreased in the edematous area adjacent to a cortical contusion [41].

### 2.1. Vasogenic Edema

Data from studies of AQP4 knock-out mice suggest that AQP4 is involved in clearing extracellular fluid from the brain parenchyma in vasogenic edema Fig. (**2**). In several models where vasogenic edema is the predominant form of edema, there is a significantly greater increase in brain water content and intracranial pressure. This has been documented in AQP4 knock-out mice compared with their wild-type counterparts, suggesting impaired brain water elimination following AQP4 deletion [31, 33]. In addition, observations in AQP4 knock-out mice with water intoxication and focal cerebral ischemia suggested that in vasogenic edema water enters the brain parenchyma independently of AQP4, but exits the brain through AQP4. This finding is intriguing, because excess fluid seems to enter the extracellular space of the brain parenchyma, but to exit using a transcellular pathway [21]. Other data hypothesized that hypertonic saline exerts its anti-edema effect by promoting an efflux of water from brain via the perivascular AQP4 pool.

### 2.2. Cytotoxic Edema

Swelling of astrocytic foot processes is a major finding in cytotoxic edema Fig. (**2**); since this is where AQP4 channels are located, the hypothesis has been advanced that they may have a role in cell swelling. AQP4 knock-out mice with ischemic stroke [22] and bacterial meningitis [32] showed decreased cerebral edema and improved outcomes. Reduced brain swelling after cerebral ischemia and water intoxication is also observed in α-syntrophin knock-out mice, which exhibit reduced AQP4 expression in astrocytic foot processes [4].

Lactic acidosis, which takes place in the brain during cerebral ischemia [37], seems to play a role in the development of cytotoxic edema [40], since lactic acid leads to swelling in cultured rat astrocytes; moreover, an increase of AQP4 expression has been demonstrated on the astrocyte cell membrane as well as AQP9 permeability [27]. In the latter case, lactate and glycerol could be cleared from the extracellular space during ischemia, with the participation of AQP9, and be used later as energy substrates. For instance, lactate has been shown to help neuronal recovery after ischemic insults [36].

### 2.3. Hydrocephalic Edema

Hydrocephalus is the result of an imbalance between cerebrospinal fluid (CSF) production and resorption, leading to an expansion of the ventricular system and to increased intracerebral pressure [42]. The latter event drives flow from the ventricles into the parenchyma, leading to extracellular edema, especially in subventricular white matter [46]. A recent study has documented that AQP4 is upregulated in periventricular white matter of hydrocephalic rats, and that up-regulation increases with disease severity, supporting an adaptive response aimed at clearing excess fluid [42].

Obstructive hydrocephalus, produced by kaolin injection into the cistern magna, induced faster ventricular enlargement in AQP4 knock-out than in wild-type mice. The diminished water permeability of ependymal layer, subependymal astrocytes, astrocytic foot processes and glia limitans produced by AQP4 deletion lessens the elimination rate of CSF through these routes [12].

Moreover AQP1 knock-out mice show a 25% reduction in the rate of CSF secretion, reduced osmotic permeability of choroid plexus epithelium, and decreased intracranial pressure [30]. These findings support a role for AQP1 in faciliting CSF secretion into the cerebral ventricles by the choroid plexus, and support the hypothesis of a role for AQP1 inhibitors in treating hydrocephalus and benign intracranial hypertension, both of which are associated with CFS formation or accumulation [28].

## 3. THERAPEUTICAL IMPLICATIONS

Data from immunostaining and protein quantification in human and animal tissues demonstrate up-regulation of AQP4 expression in primary brain tumours and stroke, and after traumatic brain injury [7, 35, 44]. Although the signaling pathways involved in disease-related expression up-regulation remain unknown, up-regulation in response to hormonal and osmotic stimulants has been demonstrated in cultured astrocytes [8, 17]. It is only a matter of time before small molecular compounds that can inhibit AQP4 function and up-regulate AQP4 expression become available, as several investigative groups have already begun testing for such compounds [12].

Drugs that target AQP4 function would provide a major therapeutic advancement in the treatment of all forms of cerebral edema, working synergistically with current therapies.

The current approach in nonsurgical treatment of edema consists primarily of systemic hypertonic fluid and corticosteroid administration, considered standard therapies of care for decades [23]. Steroids are primarily beneficial in extracellular (vasogenic) edema by suppressing inflammatory mediator release, thereby limiting BBB permeability and preventing the extracellular accumulation of edema fluid. This approach to edema is primarily preventative, and is more effective in early edema development.

In contrast, hypertonic fluids such as mannitol are effective in intracellular and extracellular edema, drawing edema fluid from the parenchyma into the vasculature, thereby enhancing the clearance of edema. Although temporarily effective in the treatment of malignant intracerebral pressure, hypertonic therapy does not prevent edema formation, and parenchymal fluid will re-accumulate after hypertonic treatment if the underlying condition is not addressed. By selectively enhancing or suppressing AQP4 activity in particular disease states, both the formation and clearance of edema from any cause can be targeted, providing effective therapy at any stage in the progression of disease.

Blocking AQP4 function, as seen in the studies of water intoxication and stroke, decreases the rate of edema formation and enhances survival. Coupled with hypertonic therapy, AQP4 inhibitors would be expected to limit the reaccumulation of edema after treatment with hypertonic fluids, making combined treatment longer lasting and more effective. Similarly, the use of AQP4 expression up-regulators would enhance edema fluid resorption in extracellular edema and could be combined with corticosteroid administration to more rapidly resolve edema associated with tumours and infection/inflammation [12].

The development of AQP4 modulating agents should be the future of molecularly targeted cerebral edema therapy.

## CONCLUSIONS

During the past two decades, the understanding of brain water physiology has progressed from the discovery of AQPs to the characterisation of their physiological and pathological functions. 

The polarized distribution of AQP4 in the perivascular astrocyte end-feet suggests that AQPs may be important in the function of the barrier as a whole, that is, endothelium plus astrocytes, although the endothelial cells themselves express at most only very low levels of AQP. This body of work suggests that AQP modulation may have several clinical uses. Although AQP modulating drugs are not currently available, studies are under way using high-throughput screening of chemical libraries to discover AQP4 modulators that could be used for treating several brain conditions including trauma, tumour, hydrocephalus and seizures.

## Figures and Tables

**Fig. (1):**
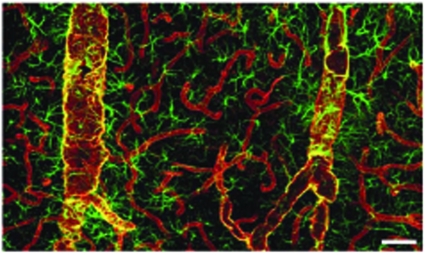
Double immunolabeling of AQP4 (red) and glial fibrillary acidic protein (GFAP) (green) in cortex. AQP4 immunolabeling reveals that the entire network of vessels, including capillaries, is covered by astrocytic processes, albeit GFAP negative. Smaller vessels and capillaries are mostly GFAP negative but display intense labeling against the astrocyte-specific channel AQP4. The AQP4 labeling reveals continuous coverage by astrocytic end feet. Figures from Simard *et al*. [38], reprinted with permission of Society of Neuroscience.

**Fig. (2):**
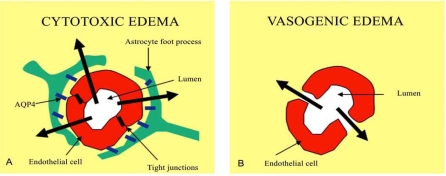
Proposed mechanism of AQP4 involvement in edema fluid formation. (**A**) In cytotoxic edema, the entry of excess fluid into the brain parenchyma is AQP4 dependent, because edema fluid flows from the vascular compartment, through intact BBB and AQP4-rich astrocyte foot processes, and accumulates primarily in astrocytes. (**B**) In vasogenic edema, water accumulation is AQP4-independent because the BBB is leaky, permitting the entry of plasma fluid directly into the brain ECS circumventing astrocyte foot processes.
