# ERRATUM

**DOI:** 10.2174/157015912800604524

**Published:** 2012-06

Due to an overlook on the author's side, the name of the first author in the article entitled as “**Aquaporin and Blood Brain Barrier**” by **Francesca Bonomini**, Rezzani R, published in the journal *Current Neuropharmacology*, 2010, Vol. 8, Issue no. 2, was wrong. The correct name is as follows.

Bonomini F, Rezzani R. Aquaporin and blood brain barrier. Curr Neuropharmacol 2010, 8(2), 92-6. 92-6

